# An exploration of immunohistochemistry-based prognostic markers in patients undergoing curative resections for colon cancer

**DOI:** 10.1186/s12885-022-09169-0

**Published:** 2022-01-14

**Authors:** Astrid Louise Bjørn Bennedsen, Luyi Cai, Rune Petring Hasselager, Aysun Avci Özcan, Khadra Bashir Mohamed, Jens Ole Eriksen, Susanne Eiholm, Michael Bzorek, Anne-Marie Kanstrup Fiehn, Thomas Vauvert F. Hviid, Ismail Gögenur

**Affiliations:** 1 Center For Surgical Science (CSS), Department of Surgery, Zealand University Hospital, Lykkebækvej 1, 4600 Køge, Denmark; 2 Cardiology department, Hospital Sønderjylland, Kresten Philipsens Vej 15, 6200 Aabenraa, Denmark; 3 Department of Pathology, Zealand University Hospital, Sygehusvej 10, 4000 Roskilde, Denmark; 4 Department of Clinical Medicine, University of Copenhagen, Blegdamsvej 3b, 2200 Copenhagen, Denmark; 5 Centre for Immune Regulation and Reproductive Immunology (CIRRI), Department of Clinical Biochemistry, Zealand University Hospital, Sygehusvej 10, 4000 Roskilde, Denmark

**Keywords:** Colon cancer, HLA-G, PD-L1, CDX2, CD3, CD8, Time to recurrence, Disease-free survival, Overall survival, Immunohistochemistry

## Abstract

**Background:**

The immune system recognizes and destroys cancer cells. However, cancer cells develop mechanisms to avoid detection by expressing cell surface proteins. Specific tumour cell surface proteins (e.g. HLA-G, PD-L1, CDX2) either alone or in combination with the relative presence of immune cells (CD3 and CD8 positive T-cells) in the tumour tissue may describe the cancer cells’ ability to escape eradication by the immune system. The aim was to investigate the prognostic value of immunohistochemical markers in patients with colon cancer.

**Methods:**

We conducted a retrospective study including patients diagnosed with pT3 and pT4 colon cancers. Immunohistochemical staining with HLA-G, PD-L1, CDX2, CD3, and CD8 was performed on tissue samples with representation of the invasive margin. PD-L1 expression in tumour cells and immune cells was reported conjointly. The expression of CD3 and CD8 was reported as a merged score based on the expression of both markers in the invasive margin and the tumour centre. Subsequently, a combined marker score was established based on all of the markers. Each marker added one point to the score when unfavourable immunohistochemical features was present, and the score was categorized as low, intermediate or high depending on the number of unfavourable stains. Hazard ratios for recurrence, disease-free survival and mortality were calculated.

**Results:**

We included 188 patients undergoing colon cancer resections in 2011–2012. The median follow-up was 41.7 months, during which 41 (21.8%) patients had recurrence and 74 (39.4%) died. In multivariable regression analysis positive HLA-G expression (HR = 3.37, 95%CI [1.64–6.93]) was associated with higher recurrence rates, while a preserved CDX2 expression (HR = 0.23, 95%CI [0.06–0.85]) was associated with a lower risk of recurrence. An intermediate or high combined marker score was associated with increased recurrence rates (HR = 20.53, 95%CI [2.68–157.32] and HR = 7.56, 95%CI [1.06–54.16], respectively). Neither high expression of PD-L1 nor high CD3-CD8 score was significantly associated with recurrence rates. Patients with a high CD3-CD8 score had a significantly longer DFS and OS.

**Conclusions:**

In tumour cells, expression of HLA-G and loss of CDX2 expression were associated with cancer recurrence. In addition, a combination of certain tumour tissue biomarkers was associated with colorectal cancer recurrence.

**Supplementary Information:**

The online version contains supplementary material available at 10.1186/s12885-022-09169-0.

## Background

Immune evasion was presented as an emerging hallmark of cancer in 2011 [1]. In the tumour microenvironment, immune cells interact continuously with the cancer cells during tumorigenesis, a process that takes several years [2, 3]. Through T-cell activation the adaptive immune system has the capacity to impair tumorigenesis, when tumour-associated antigens are presented [4]. However, the cancer cells often escape immune surveillance by activation of immune checkpoint pathways, thus avoiding anticancer immunity [5]. In recent years, immune checkpoint inhibitors have been introduced [6].

As clinical outcome varies substantially among patients diagnosed within the same tumour stage this emphasizes the need for further refinement of the current classification [7]. The Immunoscore©, which is based on the expression of cluster of differentiation 3 (CD3) and CD8 on tumour-infiltrating lymphocytes (TILs) in the tumour centre and in the invasive margin, has shown superiority as a prognostic marker over Union for International Cancer Control (UICC)-TNM classification and highlighted the importance of TILs and anti-cancer immunity [7, 8].

Several other immunohistochemical (IHC) markers are under investigation as promising prognostic or predictive biomarkers. Human leukocyte antigen G (HLA-G) is a non-classical human leukocyte antigen (HLA) class Ib molecule that has immune modulatory properties [9]. The expression of HLA-G is found in both physiological and pathological conditions [10]. HLA-G can impair the function of T-cells, B-cells, and natural killer (NK) cells through several inhibitory pathways, and is a marker of immune evasion [11–13]. Recently, HLA-G expression has been associated with a worsened prognosis in patients with colorectal cancer [14–17].

The programmed death 1 (PD-1) pathway is involved in inhibition of the immune response and the exhaustion of T-cells [18]. Programmed death-ligand 1 (PD-L1) is expressed constitutively on T-cells, B-cells, macrophages and other hematopoietic and non-hematopoietic cells, and is inducible through cytokines and in-trans binding of the immune checkpoint PD-1 [19]. Cancer cells can express PD-L1, and several published studies have investigated the role of PD-L1 both as a prognostic marker and a predictive marker for immune checkpoint blockade [6, 20–24].

Homeobox protein CDX2 (CDX2) is a marker of differentiation of colon cancer cells and has been proposed as a strong prognostic marker in patients with colon cancer [25].

The aim of this study was to explore the expression patterns of HLA-G, PD-L1, and CDX2 as well as CD3 and CD8 in a cohort of patients diagnosed with pT3 and pT4 colon cancers, and to investigate their value as prognostic markers individually and in a combined model.

## Materials and methods

### Patients

We conducted a retrospective study on archived tissue samples. The study was reported in accordance with the REMARK checklist [26]. Consecutive patients, who underwent colon cancer resection and were diagnosed with pT3 and pT4 tumours at Zealand University Hospital from 1st January 2011 until 31st December 2012, were included in the study. In the diagnostic routine setting a standardized pathological examination of the specimens had been performed according to national guidelines at the time of diagnosis. Briefly, at the macroscopic examination representative areas demonstrating key tumour features were identified and selected for paraffin embedding. Histopathological examination and tumour staging were performed according to the UICC-TNM classification. All histologic diagnoses are coded according to the Systematized Nomenclature of Medicine. Patients were searched from the records using the codes *adenocarcinoma* and *resection* combined with either *pT3* or *pT4*. Exclusion criteria were patients that were under 18 years, had a history of previous cancer, had insufficient amount of tumour tissue for the supplementary IHC stainings, were registered in the Danish Registry for Use of Tissue *(refusing to have their tissue used in research)*, had a preoperative stent, or who had received preoperative chemotherapy or radiotherapy.

### Tissue samples

Haematoxylin and eosin (H&E) stained slides from each patient were retrieved from the archive of the Department of Pathology, Zealand University Hospital, and reviewed by a consultant Pathologist. For each patient, one slide with representation of the invasive margin was selected, and the corresponding formalin-fixed paraffin-embedded (FFPE) block was retrieved for IHC stainings.

### Immunohistochemical stainings

Sections with a thickness of 4 μm were cut and slides were deparaffinised and rehydrated. Immunohistochemical stainings were performed using anti-HLA-G clone 4H84 (Exbio, Praha, Czech Republic, cat.no 11-499-C100), anti-PD-L1 clone 22C3 (Agilent/Dako, Glostrup, Denmark, cat.no M3653), anti-CDX2 clone DAK-CDX2 (Agilent/Dako, cat. no. GA080), anti-CD8 clone C8/144B (Agilent/Dako, cat. no. GA623) and anti-CD3 clone LN10 (Leica/Triolab AS, Broendby, Denmark, cat. no. NCL-L-CD3-565). All stainings was performed on the automated instrument Omnis (Agilent/Dako). For PD-L1, the protocol has been described in detail elsewhere [27]. Briefly, and for all other markers, antigen retrieval was accomplished using EnVision™ FLEX Target Retrieval Solution, High pH (Agilent/Dako, cat.no GV804) for 24 min at 97 °C. After pre-treatment, slides were incubated with the primary antibodies HLA-G (1:600), CDX2 (Ready-To-Use/RTU), CD8 (RTU) and CD3 (1:50) for 30 min at 32 °C. The reactions were detected using the standard polymer technique EnVision™ FLEX /HRP Detection Reagent (Agilent/Dako, cat. no GV800), signal intensity was enhanced using EnVision™ FLEX+ Mouse (LINKER) (Agilent/Dako, cat. no GV821) and visualized using EnVision™ Flex DAB+ Chromogen system (Agilent/Dako, cat. no. GV825) following the instructions given by the manufacturer. Finally, sections were counterstained with Haematoxylin and mounted with pertex.

### Evaluation of immunohistochemical stainings

HLA-G and CDX2 were assessed manually and semi-quantitatively. All slides were evaluated by two assessors blinded to all clinical data. At least one was a gastrointestinal pathologist. We reported HLA-G expression as either negative (< 10 positive cells) or positive (≥10 positive cells per whole slide). A positive cell was defined as cytoplasmic or membrane staining of any intensity. CDX2 expression was classified as preserved (strong positive nuclear staining in > 75% tumour cells) or reduced (< 75% tumour cells).

PD-L1, CD3 and CD8 stained tissue slides were assessed digitally and classified as high or low based on the median value of our dataset. Slides were digitized at 20x using a Leica SCN400 slide scanner (Leica Biosystems, Nussloch Germany). Algorithms for PD-L1, CD3 and CD8 stainings were developed in the TissueIA software part of Digital Image Hub (version 4.0.5) (Leica Biosystems, Nussloch Germany). The algorithms detected all intact cell nuclei based on haematoxylin counterstaining and the brown membrane DAB staining. The algorithms were adjusted and fine-tuned in close collaboration with a pathologist comparing the digital reads with manual counting until sufficient compliance was obtained.

PD-L1 was analysed as a combined positive score with percentage of all positive cells (tumour cells, lymphocytes and macrophages) divided by the total number of cells. Membrane staining in at least 75% of the membrane area were required for a cell to be classified as positive. Necrotic areas and areas of healthy tissue were excluded manually on all slides.

CD3 and CD8 expression was reported as percentages of all positive cells divided by total number of cells in the invasive margin and in the tumour centre, respectively. The invasive margin and the tumour centre was identified and delineated manually on each slide. A positive cell was defined as strong cytoplasmic staining with membranous accentuation. The median value of the percentages of CD3 and CD8 positive cells in the invasive margin and in the central tumour, respectively, was used as cut-off yielding a score of either 0 or 1. Tumours with a score of 1 for both CD3 and CD8 in the two compartments were classified as high CD3-CD8 infiltration, while tumours with any score of 0 was classified as low CD3-CD8 infiltration.

Finally, we computed a combined marker score based on features of the markers that were expected as related to immune escape by tumours. Each marker was an addend in the score with a value of zero (favourable) or one (unfavourable) depending on the expression pattern. The following unfavourable expression patterns each added one point to the score: positive HLA-G expression, low PD-L1 expression, reduced CDX2 expression, and low CD3-CD8 immune cell infiltration. The points were summarized and patients with score 0 had a low combined marker score, patients with score 1–2 had an intermediate combined marker score, and patients with score 3–4 had a high combined marker score. Patients with a low combined marker score were expected to have a favourable prognosis, while patients with a high combined marker score were expected to have an unfavourable prognosis.

**Fig. 1:**
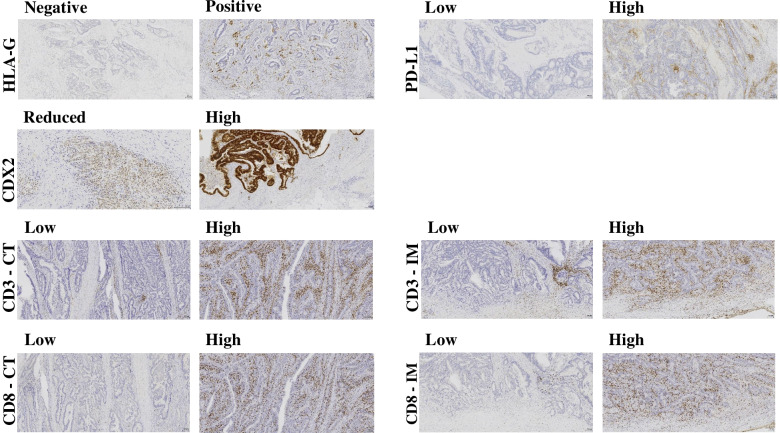
Immunohistochemical staining for HLA-G, PD-L1, CDX2, CD3 and CD8. Representative IHC stainings for negative and positive HLA-G expression, low and high PD-L1 expression, and reduced and high CDX2 expression are presented. CD3 and CD8 in the tumour centre and the invasive margin are illustrated as low and high expression, respectively. CT: tumour centre. IM: invasive margin

Figure 1 shows representative positive and negative IHC stains of all markers.

### Data collection and variables

Patient data were collected retrospectively from patient files. Baseline data consisted of age at surgery, sex, American Society of Anaesthesiologists (ASA) physical status grade, smoking status, location of primary tumour, preoperative metastases, surgery type, primary surgical procedure, 30 days postoperative complications graded by the Clavien-Dindo classification, perioperative blood transfusions, UICC stage, histological subtype, microscopic assessment of the resection margin, and information on postoperative chemotherapy. Microsatellite status, defined as either microsatellite instable (MSI) or microsatellite stabile (MSS), was collected from pathology reports, and was based on IHC for mismatch repair proteins (expression of MLH1 and MSH2, eventually combined with expression of MSH6 and PMS2 for patients with resections performed in 2012).

The primary outcome was time to recurrence defined as time in months from surgery until recurrence was recorded. Recurrence events were defined as any recorded event of clinical recurrence in the patient files. Secondary outcomes were overall survival (OS) and disease-free survival (DFS) defined as time until death or time to either recurrence or death, respectively. The end of the follow-up period was December 2017. Patients were censored at the last postoperative control for time to recurrence and DFS analyses. The patient files were linked to the Danish Central Person Registry, which ensures complete follow-up for mortality analyses.

### Statistical analysis methods

For baseline characteristics, the categorical variables were reported as number of patients and frequencies and the continuous variables as medians with inter-quartile ranges (IQR). Patients were classified according to expression of IHC markers and compared using Mann-Whitney U test for continuous variables and chi-squared test for categorical variables.

Time-to-event data were visualized using Aalen-Johansen estimates for cumulative incidence plots for recurrence and Kaplan-Meier plots for DFS and OS. Groups were compared using log-rank test for Kaplan-Meier estimates and Gray’s test for cumulative incidence, thereby accounting for mortality as a competing risk for cancer recurrence [28].

Based on existing literature and knowledge, we selected the following variables as the most important potential confounders: (< 70 or ≥ 70 years), microsatellite status (MSS or MSI), UICC stage (II, III or IV) and sidedness of tumour (right-sided or left-sided). We used multivariable Cox regression to adjust for the confounders and assessed the association of each biomarker with the outcomes separately. The variables overall met the proportional hazards assumption which was assessed by plots of Schoenfeld residuals. To account for mortality as a competing risk for recurrence, we applied the subdistribution hazards approach by Fine and Gray for these analyses [29]. Estimates are presented as hazard ratios (HR) with 95% confidence intervals (CI).

For all tests, *p*-values below 0.05 were considered statistically significant. We performed the statistical analyses using R version 3.6.1. (R Core Team (2019). R: A language and environment for statistical computing. R Foundation for Statistical Computing, Vienna, Austria. URL https://www.R-project.org/).

## Results

### Participant characteristics

**Fig. 2:**
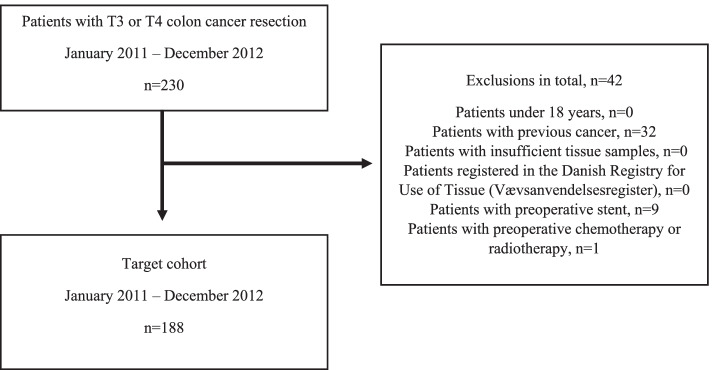
Cohort definition. A total of 188 patients were included in this study after excluding 42 patients due to the exclusion criteria

A total of 188 patients with pT3 and pT4 colon cancer tumours were included (Fig. 2). The median age at surgery was 71.5 (65–79) years, and 99 (52.7%) of the patients were females. The tumours were primarily right-sided (*n* = 103, (54.8%)) and 44 (23.4%) of the tumours were MSI. Ninety (47.9%) patients were UICC stage II, 82 (43.6%) were stage III and 16 (8.5%) stage IV. The median follow-up after resection was 41.7 (10.6–59.8) months (Table 1). During follow-up 41 (21.8%) patients experienced recurrence and 74 (39.4%) died. Eight patients did not participate in the postoperative follow-up programme, and were censored from the last outpatient visit when registering recurrence status.

**Table 1:** Baseline Characteristics. The total cohort of 188 patients with UICC stage II-IV colon cancer

	n (%)	Missing, %
N	188	
Age, years (median, IQR)	71.50 [65.00, 79.00]	0.0
Sex		
Female	99 (52.7)	0.0
Male	89 (47.3)	
ASA Score		
I	17 (11.6)	22.3
II	105 (71.9)	
III	24 (16.4)	
IV	0 (0.0)	
Tobacco		
Current smoker	33 (17.6)	0.5
Former or never smoker	154 (82.4)	
Tumour localization		
Right-sided	103 (54.8)	0.0
Left-sided	85 (45.2)	
Preoperative liver metastases		
No	174 (92.6)	0.0
Yes	14 (7.4)	
Preoperative lung metastases		
No	182 (96.8)	0.0
Yes	6 (3.2)	
Urgency		
Elective	157 (83.5)	0.0
Acute	31 (16.5)	
Procedure type		
Right hemicolectomy including transverse resection	104 (55.3)	0.0
Left hemicolectomy	76 (40.4)	
Colectomy	8 (4.3)	
Perioperative blood transfusion		
No	162 (86.6)	0.5
Yes	25 (13.4)	
Postoperative complications (Clavien-Dindo)		
0 (no complications)	126 (67.4)	0.5
1–2	13 (7.0)	
3–4	40 (21.4)	
5 (death)	8 (4.3)	
UICC stage		
II	90 (47.9)	0.0
III	82 (43.6)	
IV	16 (8.5)	
Histological type		
Adenocarcinoma NOS, high or moderate differentiated	124 (66.0)	0.0
Adenocarcinoma, poorly differentiated	32 (17.0)	
Mucinous adenocarcinoma	29 (15.4)	
Signet ring cell carcinoma	2 (1.1)	
Other carcinoma type	1 (0.5)	
Microsatellite status		
MSS	144 (76.6)	0.0
MSI	44 (23.4)	
Resection margin		
R0 (no residual tumor cells)	170 (94.4)	4.3
R1 (micro- or macroscopic residual tumor)	10 (5.6)	
Adjuvant chemotherapy		
No	107 (56.9)	0.0
Yes	81 (43.1)	
Follow-up time, months (median, IQR)	41.72 [10.56, 59.82]	0.0

Absolute numbers with percentages in parentheses unless stated otherwise

*IQR* inter-quartile range, *ASA* American Society of Anesthesiologists Physical Status Score, *UICC* Union for International Cancer Control Score, *NOS* not otherwise specified, *MSS* microsatellite stability, *MSI* microsatellite instability

### HLA-G expression status

A total of 17 (9.0%) patients were classified as HLA-G-positive (Table 2). The HLA-G-positive cancer cells were primarily located in the invasive margin or in the deeper compartments of the tumour (data not shown).

**Table 2:** Characteristics stratified according to immunohistochemistry staining results. The total cohort of 188 patients with UICC stage II-IV colon cancer and the results of immunohistochemistry (IHC) staining with the relation to variables for each marker. We used non-parametric testing to investigate differences between patients with different marker results

	HLA-G status			PD-L1 expression			CDX2 expression			CD3-CD8 infiltration		
	Negative	Positive	P	Low	High	p	Reduced	High	p	Low	High	p
N	171	17		94	94		7	181		138	50	
Age, years (median, IQR)	71.00 [65.00, 79.00]	73.00 [65.00, 80.00]	0.872	69.00 [63.25, 79.00]	73.50 [66.00, 80.00]	0.058	71.00 [65.50, 82.50]	72.00 [65.00, 79.00]	0.723	71.00 [65.00, 80.00]	72.50 [65.25, 78.00]	0.925
Sex												
Female	87 (50.9)	12 (70.6)	0.194	48 (51.1)	51 (54.3)	0.770	6 (85.7)	93 (51.4)	0.162	68 (49.3)	31 (62.0)	0.168
Male	84 (49.1)	5 (29.4)		46 (48.9)	43 (45.7)		1 (14.3)	88 (48.6)		70 (50.7)	19 (38.0)	
ASA Score												
I	16 (11.9)	1 (9.1)	0.734	8 (12.3)	9 (11.1)	0.108	1 (14.3)	16 (11.5)	0.133	14 (13.7)	3 (6.8)	0.467
II	96 (71.1)	9 (81.8)		51 (78.5)	54 (66.7)		3 (42.9)	102 (73.4)		71 (69.6)	34 (77.3)	
III	23 (17.0)	1 (9.1)		6 (9.2)	18 (22.2)		3 (42.9)	21 (15.1)		17 (16.7)	7 (15.9)	
Tobacco												
Current smoker	30 (17.6)	3 (17.6)	1.000	18 (19.4)	15 (16.0)	0.676	3 (42.9)	30 (16.7)	0.201	25 (18.2)	8 (16.0)	0.888
Former or never smoker	140 (82.4)	14 (82.4)		75 (80.6)	79 (84.0)		4 (57.1)	150 (83.3)		112 (81.8)	42 (84.0)	
Tumour localization												
Right-sided	97 (56.7)	6 (35.3)	0.151	51 (54.3)	52 (55.3)	1.000	7 (100.0)	96 (53.0)	0.039	73 (52.9)	30 (60.0)	0.485
Left-sided	74 (43.3)	11 (64.7)		43 (45.7)	42 (44.7)		0 (0.0)	85 (47.0)		65 (47.1)	20 (40.0)	
Preoperative liver metastases												
No	160 (93.6)	14 (82.4)	0.232	83 (88.3)	91 (96.8)	0.052	6 (85.7)	168 (92.8)	1.000	126 (91.3)	48 (96.0)	0.442
Yes	11 (6.4)	3 (17.6)		11 (11.7)	3 (3.2)		1 (14.3)	13 (7.2)		12 (8.7)	2 (4.0)	
Preoperative lung metastases												
No	165 (96.5)	17 (100.0)	0.951	89 (94.7)	93 (98.9)	0.213	7 (100.0)	175 (96.7)	1.000	134 (97.1)	48 (96.0)	1.000
Yes	6 (3.5)	0 (0.0)		5 (5.3)	1 (1.1)		0 (0.0)	6 (3.3)		4 (2.9)	2 (4.0)	
Urgency												
Elective	145 (84.8)	12 (70.6)	0.245	71 (75.5)	86 (91.5)	0.006	6 (85.7)	151 (83.4)	1.000	111 (80.4)	46 (92.0)	0.096
Acute	26 (15.2)	5 (29.4)		23 (24.5)	8 (8.5)		1 (14.3)	30 (16.6)		27 (19.6)	4 (8.0)	
Procedure type												
Right hemicolectomy including transverse resection	98 (57.3)	6 (35.3)	0.009	53 (56.4)	51 (54.3)	0.764	7 (100.0)	97 (53.6)	0.053	75 (54.3)	29 (58.0)	0.906
Left hemicolectomy	68 (39.8)	8 (47.1)		38 (40.4)	38 (40.4)		0 (0.0)	76 (42.0)		57 (41.3)	19 (38.0)	
Colectomy	5 (2.9)	3 (17.6)		3 (3.2)	5 (5.3)		0 (0.0)	8 (4.4)		6 (4.3)	2 (4.0)	
Perioperative blood transfusion												
No	149 (87.6)	13 (76.5)	0.359	82 (87.2)	80 (86.0)	0.977	7 (100.0)	155 (86.1)	0.622	116 (84.7)	46 (92.0)	0.289
Yes	21 (12.4)	4 (23.5)		12 (12.8)	13 (14.0)		0 (0.0)	25 (13.9)		21 (15.3)	4 (8.0)	
Postoperative complications (Clavien-Dindo)												
0 (no complications)	113 (66.5)	13 (76.5)	0.482	60 (64.5)	66 (70.2)	0.423	4 (57.1)	122 (67.8)	0.476	92 (67.2)	34 (68.0)	0.980
1–2	11 (6.5)	2 (11.8)		9 (9.7)	4 (4.3)		0 (0.0)	13 (7.2)		9 (6.6)	4 (8.0)	
3–4	38 (22.4)	2 (11.8)		19 (20.4)	21 (22.3)		3 (42.9)	37 (20.6)		30 (21.9)	10 (20.0)	
5 (death)	8 (4.7)	0 (0.0)		5 (5.4)	3 (3.2)		0 (0.0)	8 (4.4)		6 (4.4)	2 (4.0)	
UICC stage												
II	85 (49.7)	5 (29.4)	0.171	36 (38.3)	54 (57.4)	0.012	1 (14.3)	89 (49.2)	0.065	58 (42.0)	32 (64.0)	0.029
III	73 (42.7)	9 (52.9)		46 (48.9)	36 (38.3)		4 (57.1)	78 (43.1)		67 (48.6)	15 (30.0)	
IV	13 (7.6)	3 (17.6)		12 (12.8)	4 (4.3)		2 (28.6)	14 (7.7)		13 (9.4)	3 (6.0)	
Histological type												
Adenocarcinoma NOS	111 (64.9)	13 (76.5)	0.412	64 (68.1)	60 (63.8)	0.863	2 (28.6)	122 (67.4)	0.003	94 (68.1)	30 (60.0)	0.491
Adenocarcinoma, poorly differentiated	28 (16.4)	4 (23.5)		15 (16.0)	17 (18.1)		3 (42.9)	29 (16.0)		20 (14.5)	12 (24.0)	
Mucinous adenocarcinoma	29 (17.0)	0 (0.0)		14 (14.9)	15 (16.0)		1 (14.3)	28 (15.5)		21 (15.2)	8 (16.0)	
Signet ring cell carcinoma	2 (1.2)	0 (0.0)		1 (1.1)	1 (1.1)		1 (14.3)	1 (0.6)		2 (1.4)	0 (0.0)	
Other carcinoma type	1 (0.6)	0 (0.0)		0 (0.0)	1 (1.1)		0 (0.0)	1 (0.6)		1 (0.7)	0 (0.0)	
Microsatellite instability												
MSS	129 (75.4)	15 (88.2)	0.374	80 (85.1)	64 (68.1)	0.010	2 (28.6)	142 (78.5)	0.009	112 (81.2)	32 (64.0)	0.024
MSI	42 (24.6)	2 (11.8)		14 (14.9)	30 (31.9)		5 (71.4)	39 (21.5)		26 (18.8)	18 (36.0)	
Resection margin												
R0 (no residual tumor cells)	155 (94.5)	15 (93.8)	1.000	82 (94.3)	88 (94.6)	1.000	5 (71.4)	165 (95.4)	0.061	123 (93.9)	47 (95.9)	0.871
R1 (micro- or macroscopic residual tumor)	9 (5.5)	1 (6.2)		5 (5.7)	5 (5.4)		2 (28.6)	8 (4.6)		8 (6.1)	2 (4.1)	
Adjuvant chemo-therapy												
No	99 (57.9)	8 (47.1)	0.546	47 (50.0)	60 (63.8)	0.077	3 (42.9)	104 (57.5)	0.707	79 (57.2)	28 (56.0)	1.000
Yes	72 (42.1)	9 (52.9)		47 (50.0)	34 (36.2)		4 (57.1)	77 (42.5)		59 (42.8)	22 (44.0)	
Follow-up time, months (median, IQR)	47.34 [16.18, 59.98]	8.94 [3.22, 42.35]	0.009	37.85 [9.87, 59.57]	45.72 [14.56, 59.94]	0.551	6.34 [3.91, 39.10]	42.35 [12.06, 59.79]	0.217	37.57 [9.49, 59.90]	48.51 [23.70, 59.76]	0.307

Absolute numbers with percentages in parentheses unless stated otherwise. P-values are calculated using Mann-Whitney U test for continuous variables and chi square test for categorical variables

*HLA-G* human leukocyte antigen G, *PD-L1* programmed death-ligand 1, *CDX2* homeobox protein CDX-2, *CD3-CD8* cluster of differentiation 3 and 8, *IQR* inter-quartile range, *ASA* American Society of Anesthesiologists Physical Status Score, *UICC* Union for International Cancer Control Score, *MSS* micro satellite stability, *MSI* micro satellite instability

**Fig. 3:**
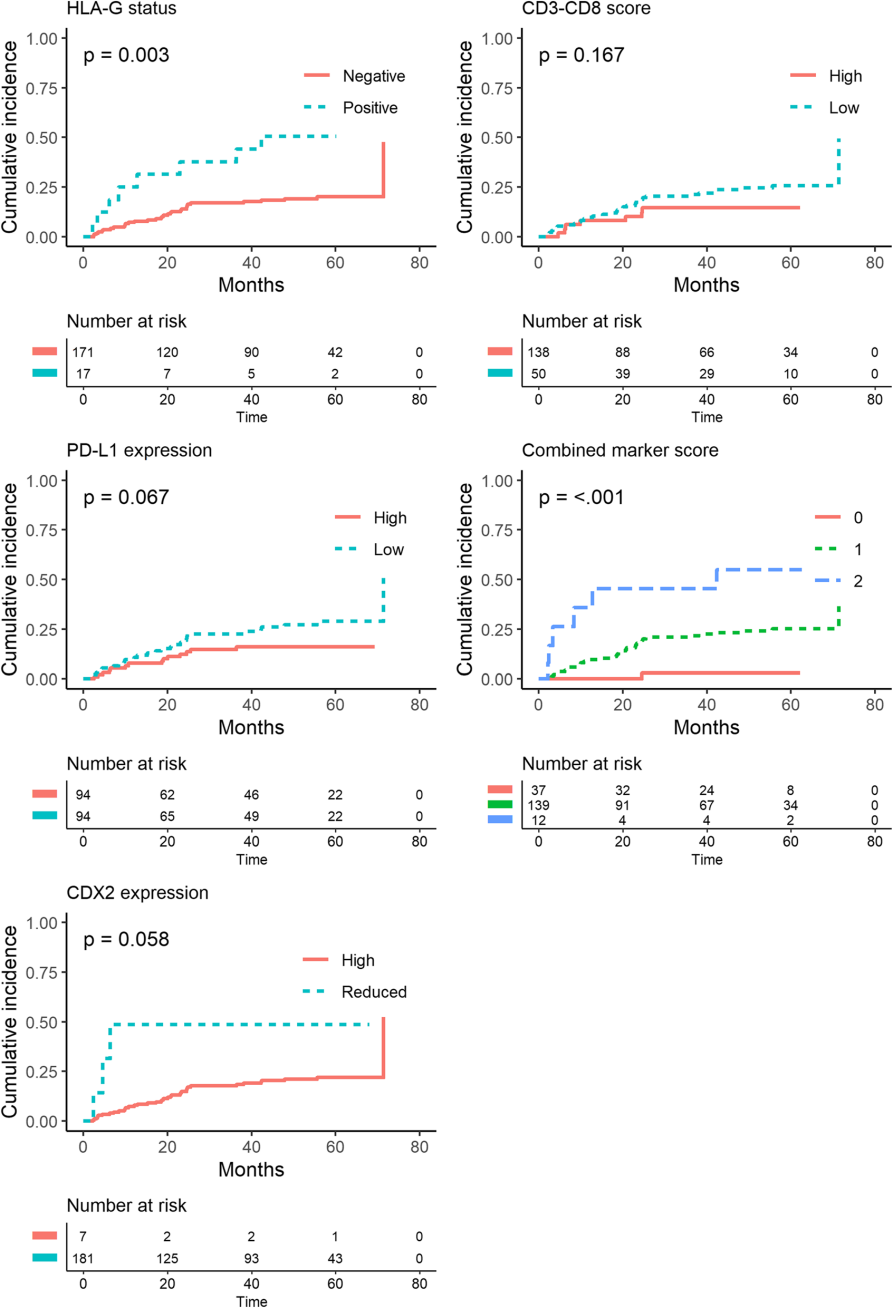
Cumulative incidence plots of recurrence. *P*-values are estimated using Gray’s test. Time-to-recurrence after colon cancer resection stratified by expression of HLA-G, PD-L1, CDX2 and the CD3-CD8 score and the combined marker score. The combined marker score was computed based on the expression of the markers. Score 0 represents a low combined marker score indicating a favourable prognosis, 1 represents an intermediate combined marker score, and 2 represents a high combined marker score indicating an unfavourable prognosis

**Fig. 4:**
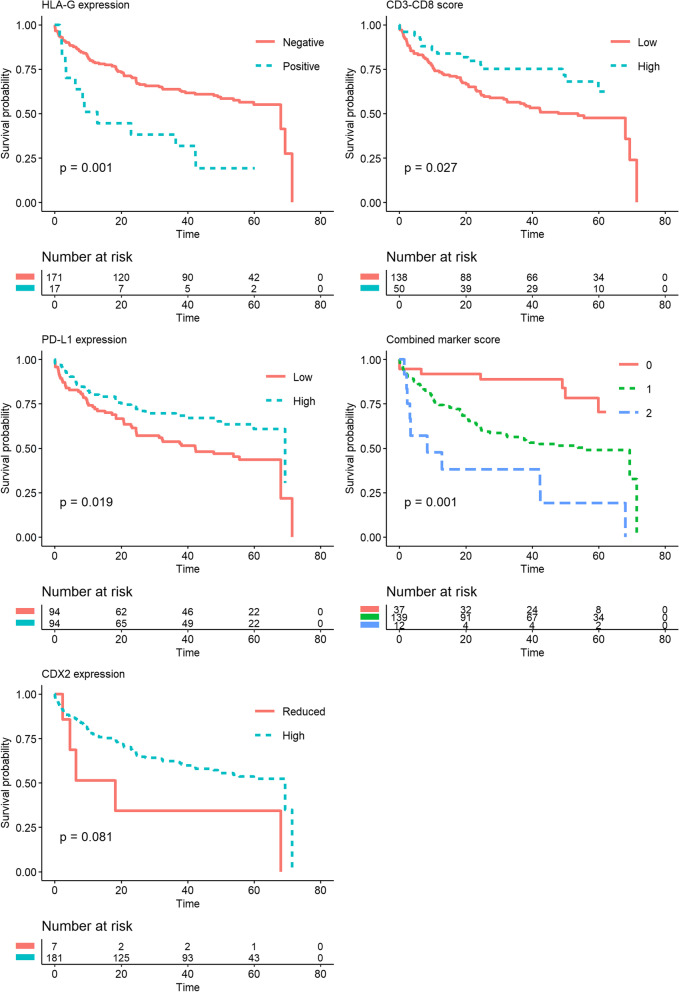
Kaplan-Meier plots of Disease-Free Survival. Disease-Free Survival (DFS) after colon cancer resection stratified by expression of HLA-G, PD-L1, CDX2, and CD3-CD8 score and combined marker score. The combined marker score was computed based on the expression of the markers. Score 0 represents a low combined marker score indicating a favourable prognosis, 1 represents an intermediate combined marker score, and 2 represents a high combined marker score indicating an unfavourable prognosis. P-values were estimated using log-rank test

**Fig. 5:**
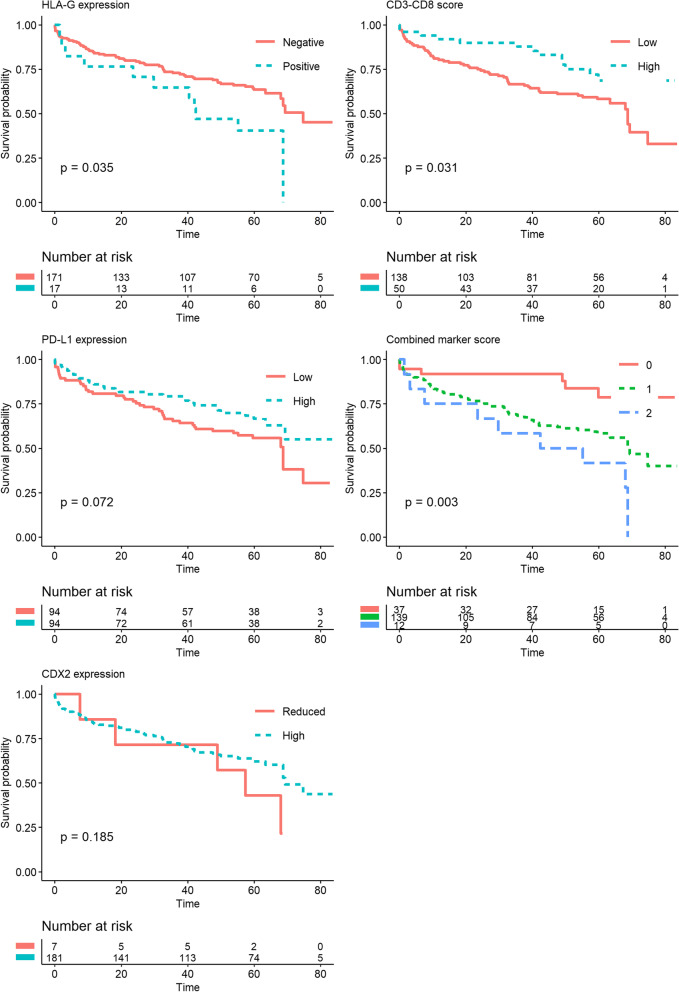
Kaplan-Meier plots of Overall Survival. Overall Survival (OS) after colon cancer resection stratified by expression of HLA-G, PD-L1, CDX2, and CD3-CD8 score and combined marker score. The combined marker score was computed based on the expression of the markers. Score 0 represents a low combined marker score indicating a favourable prognosis, 1 represents an intermediate combined marker score, and 2 represents a high combined marker score indicating an unfavourable prognosis. P-values were estimated using log-rank test

**Fig. 6:**
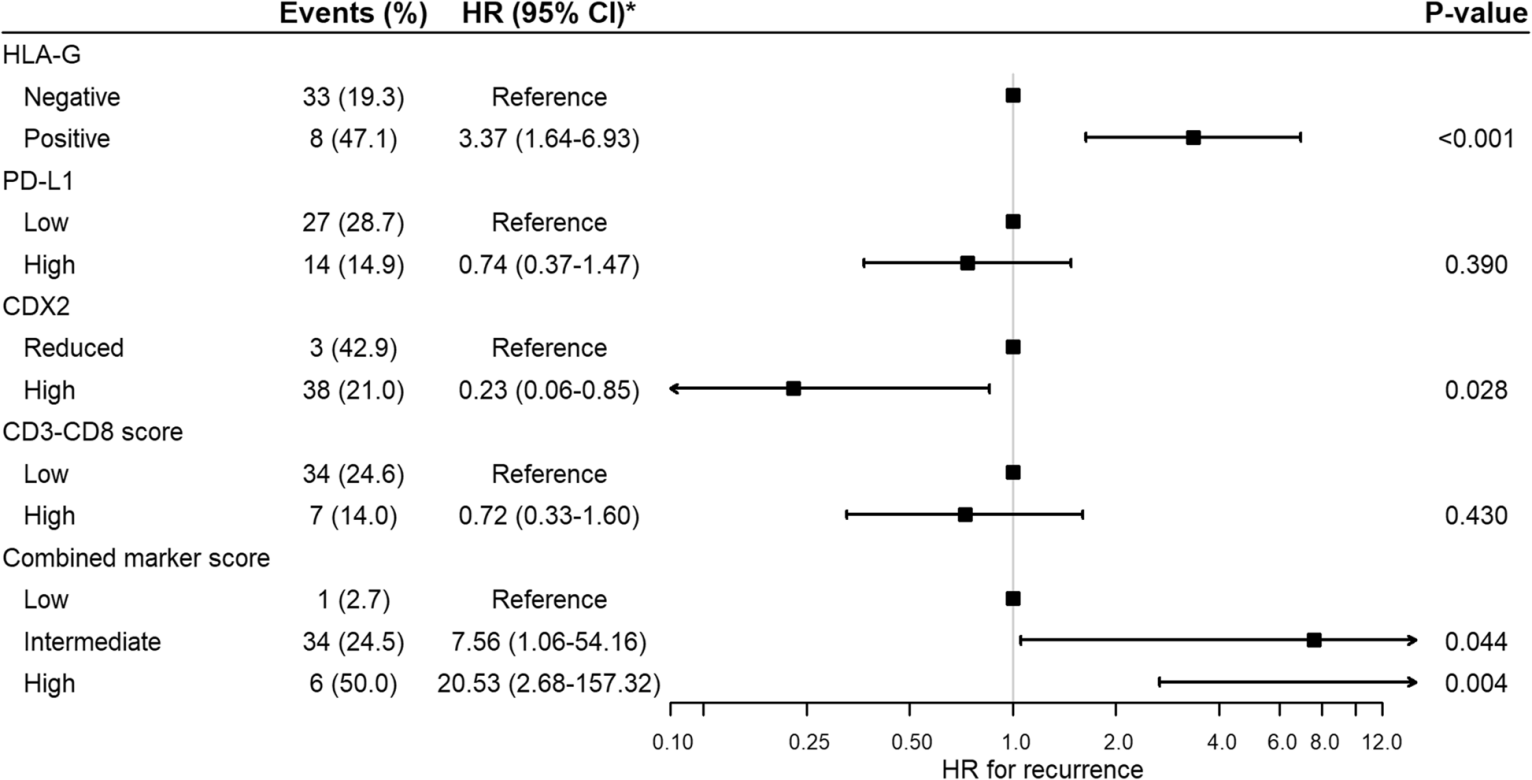
Forest plot of regression analyses of time-to-recurrence. Cox regression with subdistribution hazards approach analyses adjusted for age (< 70 or ≥ 70 years), microsatellite status (microsatellite stability or microsatellite instability), Union for International Cancer Control (UICC) stage (II, III or IV), and sidedness of tumour (right-sided or left-sided)

**Fig. 7:**
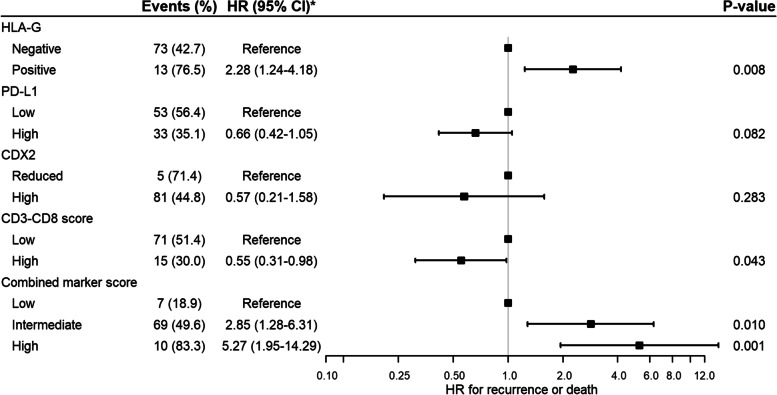
Forest plot of regression analyses of Disease-Free Survival. Cox regression analyses adjusted for age (< 70 or ≥ 70 years), microsatellite status (microsatellite stability or microsatellite instability), Union for International Cancer Control (UICC) stage (II, III or IV), and sidedness of tumour (right-sided or left-sided) was performed. During follow-up 88 (46.8%) patients had recurrence or died, and eight patients chose not to take part in the postoperative follow-up programme, and we censored them from last outpatient visit when recording recurrence

**Fig. 8:**
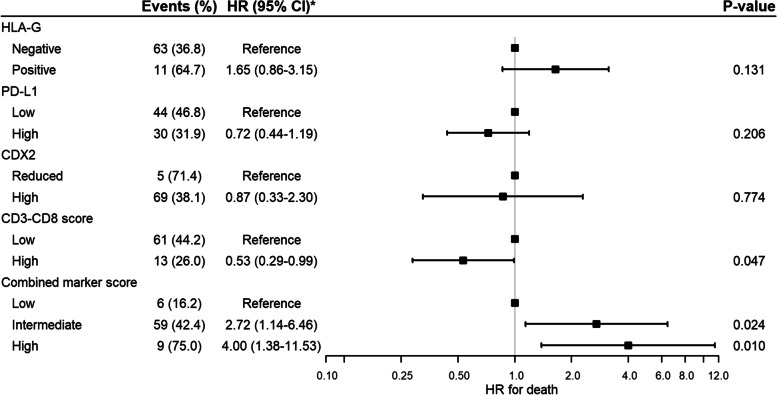
Forest plot of regression analyses of Overall Survival. Cox regression analyses adjusted for age (< 70 or ≥ 70 years), microsatellite status (microsatellite stability or microsatellite instability), Union for International Cancer Control (UICC) stage (II, III or IV), and sidedness of tumour (right-sided or left-sided) was performed. 74 (39.4%) patients died during follow-up

Of the HLA-G-positive patients, eight (47.1%) experienced cancer recurrence and 11 (64.7%) died. In the HLA-G-negative group, the death and recurrence numbers were 63 (36.8%) and 33 (19.3%), respectively. In the unadjusted non-parametric analysis there was significant difference between the groups for recurrence (*p* = 0.003, Fig. 3), DFS (*p* = 0.001, Fig. 4) and OS (*p* = 0.035, Fig. 5). Confounder adjusted multivariable regression analyses yielded higher recurrence rates, HR 3.37 (95%CI [1.64–6.93], Fig. 6), and worse DFS, HR = 2.28 (95%CI [1.24–4.18], Fig. 7), for HLA-G-positive individuals. The regression analysis for OS was not significant (HR = 1.65, 95%CI [0.86–3.15], Fig. 8).

### PD-L1 expression status

The median percentage of positive PD-L1 cells was 1.15% (IQR 0.68–2.33%) in the total cohort (Supplementary Table 1). Thirty (31.9%) patients with high PD-L1 expression were MSI, while 14 (14.9%) patients with low PD-L1 expression were MSI. A significant difference between PD-L1 expression and microsatellite status was found (*p* = 0.010, Table 2).

In the group of patients with low PD-L1 expression, 27 (28.7%) patients experienced recurrence and 44 (46.8%) patients died. In comparison, in the group with high PD-L1 expression 14 (14.9%) events of recurrence occurred, and 30 (31.9%) events of death were registered. In the non-parametric and unadjusted analyses there was no significant differences between groups for recurrence (*p* = 0.067, Fig. 3) and OS (*p* = 0.072, Fig. 5), while a significant difference was found between groups for DFS (*p* = 0.019, Fig. 4). Multivariate regression analyses adjusted for confounders yielded lower but non-significant recurrence rates in the group of patients categorized as high expression of PD-L1, HR = 0.74 (95%CI [0.37–1.47], Fig. 6). The regression analyses for DFS and OS were not significant (HR = 0.66, 95%CI [0.42–1.05] and HR = 0.72, 95%CI [0.44–1.19], respectively, Figs. 7 and 8).

### CDX2 expression status

Only seven (3.7%) patients had reduced CDX2 expression of which five were MSI and two MSS. CDX2 expression was found to be significantly different based on microsatellite status (*p* = 0.009). Three patients with reduced CDX2 expression had poorly differentiated tumours compared with 29 patients with high CDX2 expression (42.9 and 16.0%, respectively, *p* = 0.003, Table 2).

The unadjusted non-parametric analyses between groups yielded a non-significant *p*-value for recurrence (*p* = 0.058, Fig. 3), and for DFS and OS (*p* = 0.081 and *p* = 0.185, respectively, Figs. 4 and 5). High CDX2 expression was associated with a significantly lower recurrence rate, HR = 0.23 (95%CI [0.06–0.85], Fig. 6), in the confounder adjusted multivariable regression analysis. The regression analyses for DFS and OS were not significant (HR = 0.57, 95%CI [0.21–1.58] and HR = 0.87, 95%CI [0.33–2.30], respectively, Figs. 7 and 8).

### CD3 and CD8 expression status

The median percentage of CD3-positive cells in the tumour centre was 13.34% (IQR 8.46–21.05) and 18.16% (IQR 11.31–24.05) in the invasive margin. The median percentage of CD8-positive cells in the tumour centre was 6.11% (IQR 3.08–11.13) and 9.32% (IQR 5.59–14.10) in the invasive margin. The merged CD3-CD8 score yielded 138 (73.4%) low infiltrated tumours and 50 (26.6%) high infiltrated tumours (Supplementary Table 1). Eighteen (36%) patients with high infiltrated tumours were MSI, while 26 (18.8%) patients with low infiltrated tumours were MSI, and the difference was significant (*p* = 0.024, Table 2).

The unadjusted non-parametric analyses found no significant difference between groups for recurrence (*p* = 0.167, Fig. 3), while a significant difference was found for DFS and OS (*p* = 0.027 and *p* = 0.031, respectively, Figs. 4 and 5). Confounder adjusted multivariable regression analysis did not show a significant lower recurrence rate for patients with a high CD3-CD8 score (HR = 0.72, 95%CI [0.33–1.60], Fig. 6). However, this group of patients did have a significantly longer DFS, HR = 0.55 (95%CI [0.31–0.98], Fig. 7) and a significantly longer OS, HR = 0.53 (95%CI [0.29–0.99], Fig. 8).

### Combined marker score

A combined IHC score of all markers resulted in 37 (19.7%) patients with a low score, 139 (73.9%) patients with an intermediate score, and 12 (6.4%) patients with a high score (Supplementary Table 1).

In the unadjusted non-parametric analyses there were significant differences between the three groups for recurrence (*p* < 0.001, Fig. 3), DFS (*p* = 0.001, Fig. 4) and OS (*p* = 0.003, Fig. 5). Confounder adjusted multivariable regression analyses yielded a significantly higher recurrence rate for patients with an intermediate and a high combined marker score (HR = 7.56, 95%CI [1.06–54.16] and HR = 20.53, 95%CI [2.68–157.32], respectively, Fig. 6). An intermediate and a high combined marker score were associated with a significantly shorter DFS (HR = 2.85, 95%CI [1.28–6.31] and HR = 5.27, 95%CI [1.95–14.29], respectively, Fig. 7) compared with a low score. An intermediate and a high combined marker score were associated with a significantly shorter OS (HR = 2.72, 95%CI [1.14–6.46] and HR = 4.00, 95%CI [1.38–11.53], respectively, Fig. 8) compared with a low score in a confounder adjusted multivariate analysis.

## Discussion

In this study, we explored the expression of prognostic markers in patients with pT3 and pT4 colon cancers including HLA-G and PD-L1, two markers of immune evasion, as well as the expression of CDX2, a marker of differentiation, and CD3 and CD8, markers of TILs. In adjusted multivariable Cox regression models, positive HLA-G expression was associated with a shortened time to recurrence while a preserved CDX2 expression was associated with a prolonged time to recurrence. When we combined all IHC markers into a summarized score of an unfavourable expression pattern, we found an intermediate and a high combined marker score to be associated with a shortened time to recurrence.

Our results of HLA-G expression as a prognostic marker are in accordance with previously published studies on patients with colorectal cancer [14–17]. HLA-G expression has also been shown to be associated with a shortened time to recurrence, DFS and OS in several other malignancies such as gastric cancer, breast cancer, lung cancer and malignant melanoma [30–33]. During pregnancy, HLA-G modulates the maternal immune response to accept the semi-allogenic foetus [34, 35]. These results are all in accordance with a pathophysiological expression of HLA-G and its modulatory effects on cells of the immune system [10–13]. We defined HLA-G-positive tumours as 10 or more positive cells in one full slide, which may be a very low cut-off. The literature is sparse and divergent on survival analyses and cut-off values for HLA-G expression. Dichotomising HLA-G expression based on positive expression (> 0% positive cells) or a 5%-cut-off has previously been used in prognostic biomarker studies on patients with colorectal cancer [15–17, 36, 37]. We had a lower occurrence of HLA-G-positive tumours than the published studies with a > 0% cut-off with 9.0% in our cohort compared with 70.6 and 65% in two Chinese populations and 20.3% in a Dutch population, thereby in more accordance with our study [16, 17, 36]. The Dutch study utilized the same antibody (4H84) as we did while using tissue microarrays (TMAs) instead of evaluating full slides. The 4H84 mAb detects denatured HLA-G molecules. The authors included patients with colon cancer of all T-stages, although their population had primarily T3 tumours, while we in the present study only included patients with T3 and T4 colon cancer tumours [36]. The two Chinese studies both evaluated full slides; however, different anti-HLA-G antibodies were used; the MEM-G/2 mAB, which binds free heavy chain of all HLA-G isoforms, and an anti-HLA-G mAb (HGY) not available commercially that should detect both membrane and soluble HLA-G isoforms. The studies included patients with colon and rectal cancer with all T-stages. The study with the highest proportion of patients with HLA-G positive tumours did not stratify patients in colon and rectal cancer cohorts [16]. However, the other Chinese study found a lower proportion of HLA-positivity in patients with rectal cancer [17]. Direct comparison does not seem possible due to the different methods applied in these studies compared to ours e.g. TMA versus full slides and different antibodies applied. Furthermore, both inter- and intratumour heterogeneity have been reported for HLA-G in colorectal tumours [38, 39]. Thus, the expression of HLA-G varies depending on the location within the tumour. The inconsistent HLA-G findings across studies could be attributed to several factors. A number of different anti-HLA-G mAbs are used in the published studies, one (HGY) is not commercially available and staining specifities seem not to have been widely assessed. The mAbs may bind to different epitopes, which may influence the detection rate of HLA-G isoform expression in different tumours. It can be speculated that there might also be ethnic differences; the percentages of tumours expressing HLA-G are closest within the two studies including Caucasian patient groups and within the two studies including Asian patient groups, respectively. Furthermore, novel alternatively spliced HLA-G isoforms have been characterized in clear cell renal cell carcinoma specimens, which may theoretically also occur in colon cancers and influence the staining patterns [40]. Finally, even with the same population and IHC methods, formalin fixation time has been shown to affect the IHC reactions [41]. Interestingly, HLA-G may be a potential new therapeutic target for cancer immunotherapy [42]. One study utilizing chimeric antigen receptor T-cells (CAR-T cells) directed against HLA-G was recently published, while a number of patents have been filed for experimental antibodies directed against HLA-G and its receptors [43, 44].

A consensus guideline for assessment of PD-L1 has not been established for colon cancer. We used a combined positive score for cancer cells and immune cells expressing PD-L1 as a surrogate marker of immune activation. We found patients with high PD-L1 expression to have a longer DFS in unadjusted non-parametric analyses. Four studies based on TMAs have investigated the combined expression of PD-L1 in tumour and immune cells in patients with colorectal cancer [24, 45–47]. All four studies did find an association of a high combined PD-L1 expression and longer survival, however, they used different antibodies and performed manual assessment of the PD-L1 stainings. A recent meta-analysis of PD-L1 expression and prognosis in patients with colorectal cancer did not recommend PD-L1 as a prognostic marker even though the conclusion was that immune cell expression of PD-L1 was associated with a better survival [48]. As PD-L1 expression may be a marker of good prognosis when expressed by immune cells, and may be a marker of bad prognosis when expressed by tumour cells, it might be more informative not to use a combined positive score as we did, but differentiate between the cell types [23, 24]. However, our analytic platform did not allow for this distinguishment.

CDX2 is a gastrointestinal-specific transcription factor [49]. We identified only 3.7% of our cohort with a reduced CDX2 expression. Patients with reduced CDX2 expression had significantly shortened time to recurrence. Previously, loss of CDX2 has been described as strongly associated with poor prognosis in patients with colorectal cancer [25, 50, 51]. Our results support that loss of CDX2 is a marker of poorly differentiated tumours. Furthermore, we found a reduced CDX2 expression to be associated with MSI status. Interestingly, a study reported that loss of CDX2 expression could predict survival only in patients with MSS [51]. Loss of CDX2 expression has also been suggested to identify a high-risk subgroup of patients with stage II [25].

In our study, patients with a high CD3-CD8-score had a significantly prolonged DFS as well as a prolonged OS. Thus, our results are in line with those shown for the Immunoscore© in several publications and cohorts of patients with colorectal cancer [7, 8, 52]. We did not follow the Immunoscore© protocol, as we used percentages of positive cells instead of densities, different antibodies, laboratory equipment and software for digital analysis. We did, however, adopt a similar approach when calculating a score for TILs, based on digital counts of CD3- and CD8-positive cells in two tumour compartments (the tumour centre and the invasive margin). Patients with early stage disease, UICC stage I, have been found to have a higher infiltration of TILs than patients with UICC stage II-IV [53]. We did not include patients with UICC stage I, but we did, however, find patients with a high CD3-CD8 score to have a higher occurrence of UICC stage II disease than patients with a low CD3-CD8 score. We also found patients with a high CD3-CD8 score to have a higher occurrence of MSI than patients with a low CD3-CD8 score. Accordingly, tumours with MSI are associated with a high immune system activation due to the high expression of tumour-associated antigens [7].

When we combined all our markers into a combined marker score, we identified the strongest signal in the regression analyses. Both an intermediate and a high combined marker score were significantly associated with an increased risk of recurrence and mortality. Our data confirmed that a combination of prognostic markers could provide a stronger estimate of prognosis. A previous study combining the results of HLA class I- and FoxP3-expression based on a computed immune phenotype, could identify a distinct survival pattern between three different phenotypes [36]. The width of the 95% CI in our study, reveals that our HR should be interpreted with great care. When calculating the score, all markers contributed with the same weight to the total score. However, this may not be the optimal approach as each marker may contribute differently to the risk of recurrence or death.

A strength of this study is inclusion of consecutive patients during a two-year inclusion span. We chose to focus on patients with pT3 and pT4 tumours in the colon based on the higher risk of recurrence [54]. In all tumour samples, the invasive margin was represented and assessment was performed on full slides. We investigated the expression of more than one immune checkpoint in patients with colon cancer, and each patient was analysed for TILs. Apart from the previously mentioned limitations only a low number of patients with reduced CDX2 expression (*n* = 7) and positive HLA-G expression (*n* = 17) were identified. This resulted in limited statistical power in our analyses. We saw the strongest signal in the time-to-recurrence analyses. Time-to-recurrence comes with a threat of competing risk bias in case patients die before they can develop recurrence [55]. We attempted to reduce this risk by applying the Fine-Gray method.

## Conclusions

In conclusion, we investigated HLA-G, PD-L1, CDX2, and CD3 and CD8 as prognostic markers in patients with pT3 and pT4 colon cancers. We found positive HLA-G expression, and a high combined marker score to be independently associated with a shortened time to recurrence. Preserved expression of CDX2 was independently associated with a longer time to recurrence.

## Supplementary Information

**Additional file 1 **: **Supplementary Table 1**. PD-L1, CD3 and CD8 analyses and calculation of scores.

## Data Availability

The datasets used and analysed during the current study are available from the corresponding author on reasonable request.

## Abbreviations

ASA: American Society of Anaesthesiologists
CAR-T cells: Chimeric antigen receptor T-cells
CD3: Cluster of differentiation 3
CD8: Cluster of differentiation 8
CI: Confidence intervals
DFS: Disease-free survival
FFPE: Formalin-fixed paraffin-embedded
H&E: Haematoxylin and eosin
HLA: Human leukocyte antigen
HLA-G: Human leukocyte antigen G
HR: Hazard ratios
IHC: Immunohistochemical
IQR: Inter-quartile ranges
MSI: Microsatellite instable
MSS: Microsatellite stabile
NK cells: Natural killer cells
NOS: Not otherwise specified
OS: Overall survival
PD-1: Programmed death 1
PD-L1: Programmed death-ligand 1
TILs: Tumour-infiltrating lymphocytes
UICC: Union for International Cancer Control
