# Supplementary material for: An exploration of immunohistochemistry-based prognostic markers in patients undergoing curative resections for colon cancer
Source: BMC Cancer. 2022 Jan 14;22:62. doi: 10.1186/s12885-022-09169-0 (PMC8759288; doi:10.1186/s12885-022-09169-0)
Supplement: Supplementary file 1 — Additional file 1 : Supplementary Table 1. PD-L1, CD3 and CD8 analyses and calculation of scores. [file 12885_2022_9169_MOESM1_ESM.docx]

| ***Supplementary Table 1.*** | PD-L1, CD3 and CD8 analyses and calculation of scores | |
| --- | --- | --- |
|  | Total cohort (n=188) | |
| PD-L1 |  |  |
| %Positive PD-L1 cells, median (IQR) | 1.15 | (0.68-2.33) |
|  |  |  |
| CD3 |  |  |
| %Positive CD3 cells in CT, median (IQR) | 13.34 | (8.46-21.05) |
| %Positive CD3 cells in IM, median (IQR) | 18.17 | (11.31-24.05) |
|  |  |  |
| CD8 |  |  |
| %Positive CD8 cells in CT, median (IQR) | 6.11 | (3.08-11.13) |
| %Positive CD8 cells in IM, median (IQR) | 9.32 | (5.59-14.10) |
|  |  |  |
| Sum of CD3 and CD8 scores |  |  |
| *0, n (%)* | 48 | (25.5%) |
| *1, n (%)* | 25 | (13.3%) |
| *2, n (%)* | 45 | (23.9%) |
| *3, n(%)* | 20 | 10.6%) |
| *4, n(%)* | 50 | (26.6%) |
|  |  |  |
| Combined marker scores summarized |  |  |
| *0, n (%)* | 37 | (19.7%) |
| *1, n (%)* | 58 | (30.9%) |
| *2, n(%)* | 81 | (43.1%) |
| *3, n(%)* | 12 | (6.4%) |
| *4, n(%)* | 0 | (0%) |

**Supplementary Table 1:** PD-L1, CD3 and CD8 analyses and calculation of scores. PD-L1, CD3 and CD8 were reported as percentages of positive cells divided by total number of cells. Both tumour cells and immune cells were included when counting PD-L1-positive cells. CD3 and CD8 expression was counted in two separate compartments; the invasive margin and the tumour centre. The median percentage for each lymphocyte subpopulation and compartment was used as cut-off values yielding a score of either 0 or 1. Tumours with a score of 1 for CD3 and CD8 in all compartments were classified as high CD3-CD8 infiltration, while tumours with any score of 0 was classified as low CD3-CD8 infiltration. We computed a combined marker score: HLA-G expression (positive +1, negative 0), PD-L1 expression (low +1, high 0), CDX2 expression (reduced +1, high 0), and CD3-CD8 score (low +1, high 0). The points were summarized and patients with score 0 had a low combined marker score, patients with score 1-2 had an intermediate combined marker score, and patients with score 3-4, had a high combined marker score indicating an unfavourable prognosis. IQR: Interquartile range. CT: tumour centre. IM: invasive margin.
